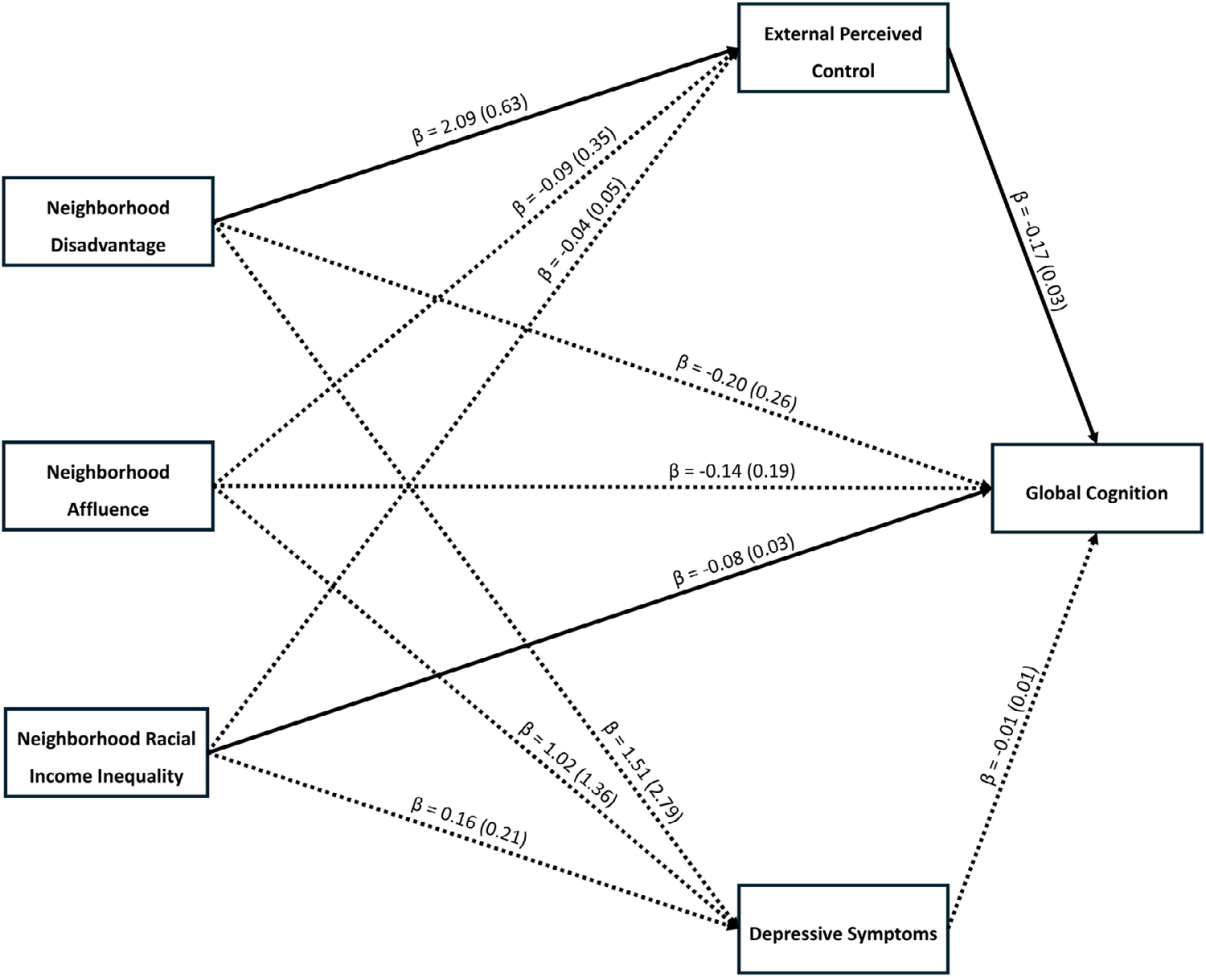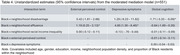# Psychological pathways linking neighborhood socioeconomic factors to cognitive health in Black and White older adults

**DOI:** 10.1002/alz70860_100289

**Published:** 2025-12-23

**Authors:** Laura B. Zahodne, Emily P. Morris, Robrielle M. Pierce, Ketlyne Sol, Kiana A. Scambray, Monica E. Walters, Lauren Taylor, Vivian Ku, Sofia Lomba, Noah Green, Philippa Clarke

**Affiliations:** ^1^ University of Michigan, Ann Arbor, MI, USA

## Abstract

**Background:**

Both the level and distribution of neighborhood‐level socioeconomic resources are associated with cognitive health in later life. This study examined psychological mechanisms underlying these associations for Black and White older adults.

**Method:**

Data from 591 (50% Black, 43% White) older adults from the Michigan Cognitive Aging Project were linked to census tract‐level information on neighborhood disadvantage, affluence, and racial income inequality from the National Neighborhood Data Archive. Global cognition was a z‐score composite of five domains from a comprehensive neuropsychological battery. Psychological mediators were external perceived control and depressive symptoms measured with self‐report questionnaires. Simultaneous mediation models accounting for neighborhood clustering examined associations between neighborhood factors and cognition through external perceived control and depressive symptoms.

**Result:**

External perceived control, but not depressive symptoms, mediated the negative association between neighborhood disadvantage and cognition in the whole sample. Moderated mediation models showed a stronger neighborhood disadvantage‐cognition association independent of the psychological mediators among Black participants and stronger associations between affluence and both psychological mediators among White participants.

**Conclusion:**

Psychosocial stress may be one pathway linking neighborhood disadvantage to dementia risk among diverse older adults. Future studies should characterize additional modifiable pathways, particularly for Black older adults, who also live in neighborhoods with greater disadvantage than White older adults, on average. Future studies should also investigate why neighborhood affluence may have stronger positive psychological and cognitive effects among White older adults than Black older adults, which could involve discrimination and racially patterned barriers to accessing neighborhood resources.